# Quantification of Maternal Serum Cell-Free Fetal DNA in Early-Onset Preeclampsia

**DOI:** 10.3390/ijms14047571

**Published:** 2013-04-08

**Authors:** Hong Yu, Yanting Shen, Qinyu Ge, Youji He, Dongyan Qiao, Mulan Ren, Jianqiong Zhang

**Affiliations:** 1 Department of Obstetrics and Gynecology, Zhongda Hospital, Medical School, Southeast University, Nanjing 210009, China; E-Mails: shenyanting798@126.com (Y.S.); qiaodongyanqq@163.com (D.Q.); renmulan@seu.edu.cn (M.R.); 2 State Key Laboratory of Bioelectronics, Southeast University, Nanjing 210096, China; E-Mail: geqinyu@seu.edu.cn; 3 Department of Microbiology and Immunology, Medical School, Southeast University, Nanjing 210009, China; E-Mail: heyouji@yahoo.com

**Keywords:** preeclampsia, cell-free fetal DNA, HCG

## Abstract

The aim of this study was to determine whether the increased serum cell-free fetal DNA (cffDNA) level of gravidas developed into early-onset preeclampsia (EOPE) subsequently in the early second trimesters is related to prenatal screening markers. Serum was collected from 1011 gravidas. The level of cffDNA and prenatal screening markers were analyzed in 20 cases with EOPE and 20 controls. All fetuses were male. The maternal serum cffDNA level was assessed by amplification of the Y chromosome specific gene. Correlations between the variables were examined. (Logged) cffDNA in EOPE (median, 3.08; interquartile range, 2.93–3.68) was higher than controls (median, 1.79; interquartile range, 1.46–2.53). The increased level of (logged) cffDNA was correlated significantly with the increased human chorionic gonadotropin (HCG) level (*r* = 0.628, *p* < 0.001). Significant reciprocal correlations between cffDNA and babies’ birth weight as well as gestation weeks at delivery were noted (*r* = −0.516, *p* = 0.001; *r* = −0.623, *p* < 0.001, respectively). The sensitivity and specificity of cffDNA to discriminate between the EOPE cases and the controls were 90% and 85%, respectively. CffDNA is a potential marker for EOPE, which had a significant reciprocal correlation with babies’ birth weight and gestation weeks at delivery. Moreover, it may help in indicating the underlying hypoxic condition in the placenta.

## 1. Introduction

Preeclampsia (PE) is multisystem disorder in the second and third trimesters. PE is unique to pregnancy and it is one of the leading causes of maternal and fetal/neonatal morbidity and mortality worldwide [1]. Its clinical findings can manifest as a maternal syndrome (hypertension and proteinuria with or without other multisystem abnormalities) and/or a fetal syndrome (fetal growth restriction (FGR), reduced amniotic fluid, and abnormal oxygenation) [2]. PE can be classified as early-onset preeclampsia (EOPE) and late-onset preeclampsia according to its onset time. It is diagnosed as EOPE, when the PE onset is earlier than 34th gestation weeks. Although EOPE has raised more and more attention in recent years, no reliable criteria or methods are available for early prediction of EOPE [3]. Once it is detected, mothers and infants have been subject to varying degree of damage. Therefore, there is a need for widely applicable and affordable tests which can identify women at risk early in pregnancy and subsequently monitor them throughout pregnancy, and thus provide the best prenatal care for patients and their children.

Recently, more and more attentions have turned towards developing non-invasive testing methods, including ultrasound examination and the quantification of various blood-borne and urinary biomarkers. The discovery of cell-free fetal (cff) DNA in maternal blood opened a new perspective in the field of non-invasive prenatal diagnosis both in research and clinical care [4]. To date, two applications of cffDNA, namely gender determination for X-linked genetic disorders and the prenatal diagnosis of the fetal Rhesus D status, have already translated into clinical routine [5–7]. Furthermore, several researches have indicated an increase of circulating cffDNA in maternal plasma under certain pathologic conditions during pregnancy, such asPE [8,9]. Several studies reported that the maternal plasma or serum concentrations of cffDNA from pregnant women with PE were significantly higher than those from normotensive control subjects [10–25]. It has been postulated that impaired trophoblastic invasion of the maternal spiral arteries leads to placental ischemia, with release into the maternal circulation of necrotic or apoptotic syncytiotrophoblast fragments that contain fetal DNA [22,23]. In addition to evidence for increased entry of cffDNA into the maternal circulation, there is also evidence that, in PE, there is reduced clearance of cffDNA from maternal plasma [14]. However, there is controversy as to whether the altered levels precede the onset of the disease [20–24]. In our study, we conducted a prospective study and collected maternal serum in their early second trimesters to quantify cffDNA to explore whether it could be used to predict the development of EOPE before the onset of symptoms in patients at risk, and to assess the possibility of it in predicting EOPE.

Recent data showed that alpha-fetoprotein (AFP) and human chorionic gonadotropin (HCG), both are serum prenatal screening markers in second trimesters for birth defects, could predict PE as well [26,27]. We, therefore, examined the possible association between cffDNA and the two prenatal screening markers.

## 2. Results and Discussion

### 2.1. Results

#### 2.1.1. Maternal Epidemiological Characteristics

One thousand and eleven subjects were interviewed by telephone and all of them finished the epidemiological survey questionnaires. Among them, 30 cases developed into EOPE subsequently. The incident of it was 2.97%, which was similar to the incidence reported previously. Eight hundred and sixty seven pregnant women were normal; they did not experience any complications and resulted in the live birth of phenotypically normal neonates. The maternal characteristics are shown in Table 1. In the EOPE group, compared with the control group, the maternal age and pregestational body mass index (BMI) were higher, and more women had the family history of hypertention, previous abortion, and the medical history of hypertension, diabetes and nephritis. These findings were consistent with the previous studies [28].

#### 2.1.2. The Levels of Maternal Serum cffDNA, HCG and AFP in the EOPE and Control Groups

**Figure 1:**
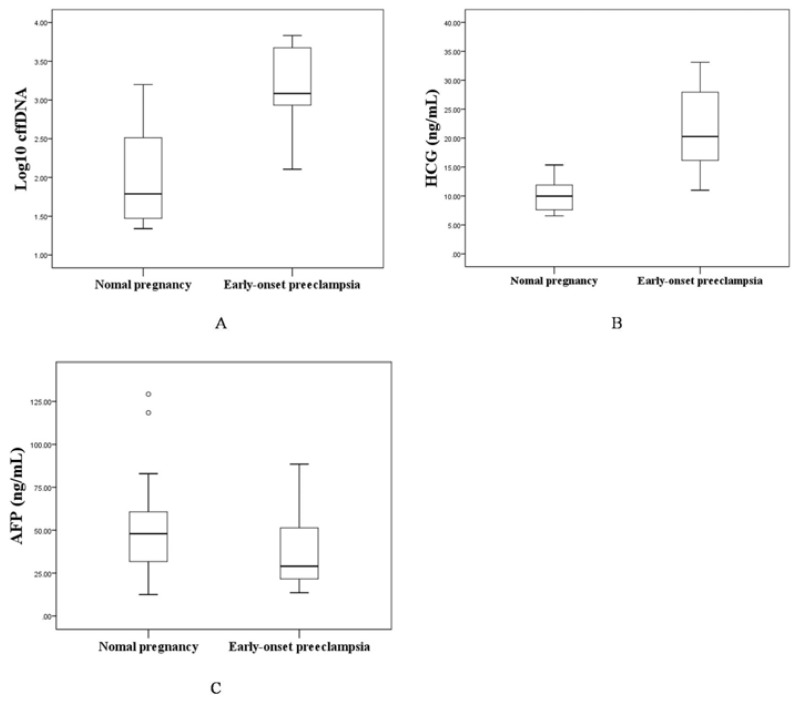
(**A**) box plot of (logged) cell-free fetal DNA (cffDNA) in the control and early-onset preeclampsia (EOPE) groups; (**B**) box plot of human chorionic gonadotropin (HCG) in control and EOPE groups; (**C**) box plot of alpha-fetoprotein (AFP) in control and EOPE groups.

The levels of maternal serum cffDNA, HCG and AF*P* were analyzed in 40 pregnant women who were carrying male fetuses (Table 2 and Figure 1). The medians of (logged) cffDNA and HCG were significantly higher in pregnant women with EOPE than in controls (median: 3.08, interquartile range: 2.93–3.68 *vs.* median: 1.79, interquartile range: 1.46–2.53, *p* < 0.001; median: 20.27 ng/mL, interquartile range: 16.05–28.12 ng/mL *vs.* median: 9.98 ng/mL, interquartile range: 7.52–12.04 ng/mL, *p* < 0.001). However, there was no statistical difference in the level of maternal serum AF*P* between the two groups (median: 29.00 ng/mL, interquartile range: 21.42–52.43 ng/mL *vs.* median: 47.94 ng/mL, interquartile range: 31.36–61.85 ng/mL, *p* = 0.094).

#### 2.1.3. The Correlation between the Levels of cffDNA and Prenatal Screening Markers

**Figure 2:**
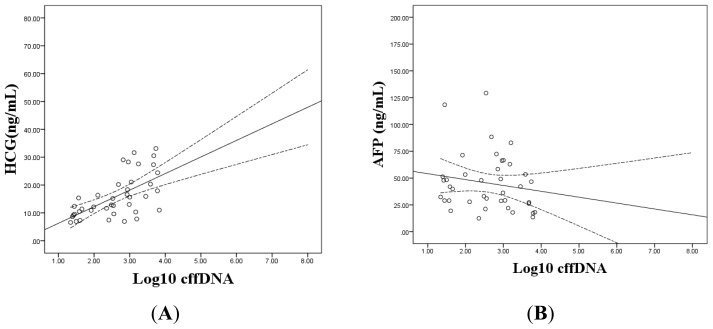
The correlation between (logged) cffDNA and HCG was shown in (**A**). The correlation between (logged) cffDNA and AFP was shown in (**B**). The lines display the regression coefficient with 95% confidence for the mean.

We further analyzed the correlation between the levels of (logged) cffDNA and prenatal screening markers (HCG and AFP). The significant positive correlation was noted between (logged) cffDNA and HCG (*p* < 0.001, *r* = 0.628), but no statistical correlation between (logged) cffDNA and AF*P* was found (Figure 2).

#### 2.1.4. The Evaluation of the Value of cffDNA in Predicting EOPE

**Figure 3:**
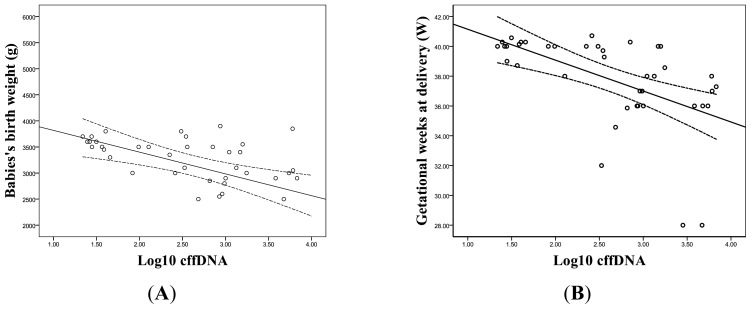
The correlation between (logged) cffDNA and babies’ birth weight was shown in (**A**). The correlation between (logged) cffDNA and gestational weeks at delivery was shown in (**B**). The lines display the regression coefficient with 95% confidence for the mean.

**Figure 4:**
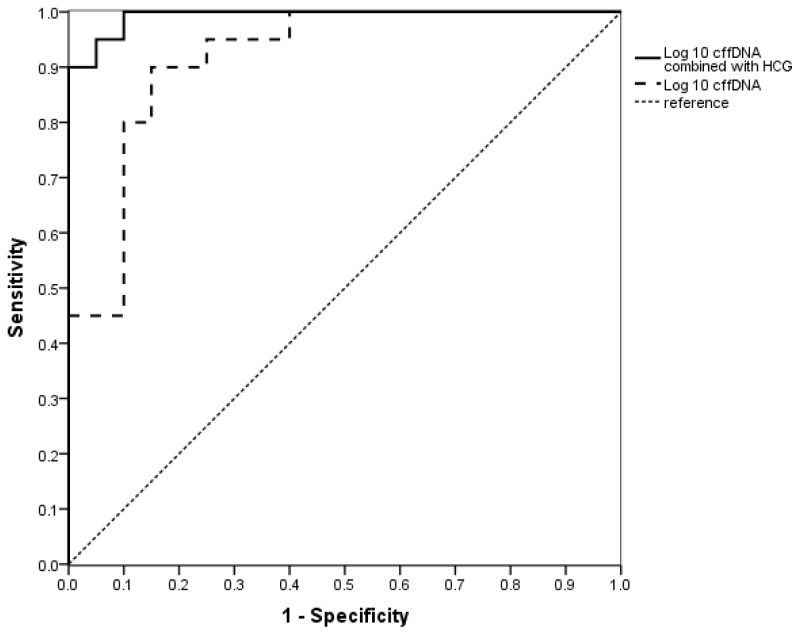
The receiver operating characteristic (ROC) curves of cffDNA alone and cffDNA combined with HCG to predict EOPE were shown.

In order to futher evaluate the value of cffDNA in predicting EOPE, the correlations of the level of (logged) cffDNA with gestation weeks at delivery and babies’ birth weight were analyzed respectively. Significant reciprocal correlations were noted: *r* = −0.623, *p* < 0.001 and *r* = −0.516, *p* = 0.001, respectively (Figure 3). A cut-off value of (logged) cffDNA at 2.62 was chosen by ROC curve analysis, which provided a sensitivity of 90% and a specificity of 85% to discriminate between the cases at risk for EOPE and the normal controls. We further found that the sensitivity and specificity were better when using cffDNA and HCG together as a predicting marker than those of using cffDNA alone. When the cut-off value was 0.05, the sensitivity and specificity were 95% respectively. (Figure 4).

### 2.2. Discussion

Recent researches showed that the level of serum cffDNA was significantly higher in PE mothers than that in the controls. Moreover, the increased cffDNA may be the consequence of both exaggerated shedding of placental apoptotic and/or necrotic materials triggered by fetoplacental hypoxemia and decreased clearance from maternal blood [14,29]. In our study, we had the same findings. Apart from this, we also found that it had good sensitivity of 90% and specificity of 85% in predicting EOPE when the cut-off value of (logged) cffDNA was 2.62. It may help in prompting the maternal-fetal pregnancy outcomes. The higher cffDNA level was associated with, the lower babies’ birth weight and the shorter gestation weeks at delivery. Since the placenta is one of the major sources of cffDNA in pregnancy, our observation also implies that the circulating cffDNA may help in understanding the biological nature of EOPE, an underlying hypoxic condition in the placenta.

The measurement of the cffDNA concentration in maternal plasma or serum may have diagnostic importance in PE pregnancies. The use of cffDNA for noninvasive prenatal diagnosis is expanding after robust data regarding the identification of specific fetal genes including RhD and Y chromosome specific genes [4]. The introduction of real-time quantitative PCR has increased its clinical role of cffDNA because of the high sensitivity and specificity and fewer problems with contamination. The Y chromosome DNA measurement we used in this study relates solely to fetal-derived material in contrast to other metabolic or hormonal markers for PE in maternal blood, which includes a mixture of maternal and fetal origin. However, it is only applicable to pregnancies with male fetuses and future research needs to identify markers that would allow monitoring of all pregnancies. Recently, studies showed that hypermethylated RASSF1A gene can be used as an epigenetic marker to detect the fetal DNA in maternal plasma, which may be expanded to the clinical application of gender independent noninvasive prenatal diagnosis [8].

In addition, our study revealed that the level of maternal serum HCG in the early second trimesters increased significantly in the women with EOPE compared to the controls. HCG, one of the major biomarkers of prenatal screening, consisting of β and α subunits, is a kind of glycoprotein secreted by placenta syneytiotrophoblasts. It could be used to predict the onset of EOPE. PE is a placenta derived disease, with a series of pathophysiological changes before clinical symptoms, such as reactive hyperplasia of syneytiotrophoblasts caused by placental hypoxemia which could increase the HCG secretion [30–35]. Jin *et al.*[34] conducted a prospective cohort study. They tested 3076 gravidas and found that the level of serum HCG in 32 gravidas ultimately experienced PE was significantly higher than that in normal pregnancies. This finding was consistent with ours. Since both cffDNA and HCG were elevated prior to the onset of EOPE, we compared the levels of these two parameters by the Spearman rank test and found that there was a significant positive correlation between them, which supports the hypothesis that there was an underlying hypoxic condition in the placenta in PE before the clinical syndromes manifest. This is the first study to show that the increased cffDNA concentration in serum samples from pregnant women at-risk for EOPE correlated strongly with the increased HCG concentration in those samples. In addition, we found that the sensitivity and specificity were further improved when using cffDNA together with HCG as a predicting marker for EOPE. Another major biomarker of prenatal screening is AFP, a kind of embryo-specific globulin. It is generated by yolk sac and hepatocytes in embryonic period. After the 12th gestation week, liver becomes the major organ for AF*P* production. Under normal conditions, only a small amount of AF*P* could cross the placenta into maternal circulation. Some research showed that the level of AF*P* in severe PE patients increased during late trimester of pregnancy and pointed that the increased level of AF*P* in pregnancies with PE may be related to the increase of placenta and fetal membrane permeability caused by insufficient reconstruction of spiral arteries and hypoxemia [12]. However, our study showed no obvious difference between pregnant women developed into EOPE subsequently and controls (*p* = 0.162), which is consistent with the previous research [34]. Therefore, we presumed that the level of serum AF*P* might be a hint for severity of PE, but not for its early prediction.

## 3. Experimental Section

### 3.1. Study Population

This was a prospective study in singleton pregnancies. 1011 pregnant women visiting Prenatal Diagnosis Center of Zhongda Hospital affiliated to Southeast University from November 2010 to January 2011 at 15 to 20^+6^ gestation weeks were recruited for the study. Their serum were collected and stored at −70 °C for subsequent biochemical analysis. Written informed consent was obtained from the women who agreed to participate in the study, which was approved by the institutional review board at Zhongda Hospita. All of them were interviewed by telephone about the pregnancy outcome, and asked to complete a questionnaire on personal essential information, history of present illness (urinary tract infection, periodontal diseases, present diseases), history of past illness (history of hypertension, diabetes, kidney diseases, polycystic ovarian syndrome, PE), history of pregnancy (last menstrual period, age of menarche, history of delivery, history of spontaneous abortion), family history (diabetes, hypertension, cardiovascular and cerebrovascular diseases), smoking history, progestational body mass index (BMI), *etc.* 30 pregnant women developed into EOPE subsequently, and 867 pregnant women who did not experience any complications and resulted in the live birth of phenotypically normal neonates were grouped to control group. We measured cffDNA in samples from 40 cases carrying male fetus, including 20 cases with EOPE and 20 control pregnant women, who were matched in maternal age and gestation weeks at enrollment. We collected the data of HCG and AF*P* directly from the Prenatal Diagnosis Center of Zhongda Hospital affiliated to Southeast University.

The definition of EOPE includes: PE onset is earlier than 34th gestation weeks [3]; the development of diastolic blood pressure of ≥90 mm Hg on at least two occasions 4 h apart after 20 weeks of gestation in previously normotensive women and proteinuria of ≥300 mg in 24 h or two readings of at least “++” on dipstick analysis of midstream or catheter urine specimens if no 24-h collection is available [36]. Intrauterine growth restriction (IUGR) refers to a birth weight that is below the 10th percentile.

### 3.2. Serum DNA Extraction

Maternal serum in the second trimesters was provided by the Prenatal Diagnosis Center of Zhongda Hospital affiliated to Southeast University. 100 μL maternal serum was incubated at 56 °C for 10 min with 200 μL enzyme reaction solution (1% SDS; 5 mM EDTA-Na_2_; 10 mM Tris-HCl) and 5 μL 20 mg/mL Proteinase K (TaKaRa, Japan), and precipitated at room temperature for at less 30 min by using 300 μL NaI solution and 600 μL isopropyl alcohol, and then centrifuged (25,000× *g* for 20 min) to remove the supernatant. The sediment was rinsed by 40% isopropyl alcohol and 70% alcohol relatively. Finally, DNA was dissolved in 20 μL volumes of TE solutionand stored at −70 °C. The genomic DNA extracted from the health man was isolated from blood using the QIAamp DNA Mini Blood Kit (Qiagen Inc., Valencia, CA, USA).

### 3.3. Real-Time Quantitative PCR Amplification

The Y chromosome specific gene was used to determine the level of cffDNA by SYBR GREEN_fluorescence quantitative real-time PCR assay (FQ-PCR) using BioRad Chorom 4 detection system. The sequences of the primers were designed as follow: upstream primer was GTCATAGAAGAGTCAAGTCAGTCA; downstream primer was ACAAGGGAACTGATATCCAG TGGCGAC. The reactions were set up in a total volume of 20 μL, containing 1 μL DNA, 10 μL SYBR^®^*Premix Ex Taq*™ (TaKaRa, Japan), 1 μL of 10 μM upstream and downstream primer respectively and 7 μL sterilized ultrapure water.

PCR was performed using the following programs: 95 °C for 2 min, followed by 40 cycles of 95° for 15 s and 60° for 30 s. To determine the copy number of cffDNA in the serum sample, a linear regression curve using the DNA extracted from the health man and diluted from 1:10 to 1:10^6^ was employed. The standard preparation and each cffDNA sample were tested on the same reaction tubes for each PCR run, and each concentration was tested in at least three replicates, the results’ outliers were excluded, and the rest results were averaged. Negative reaction and blank control were included in every analysis to minimize the risk of contamination.

### 3.4. Statistical Analysis

The SPSS statistical software package (version 13.0; SPSS Inc, Chicago, IL, USA) was used for all data analyses. The normality of the distributions was confirmed by the Kolmogorov-Smirnov test. Distributions of demographic characteristics were analyzed by the Student t test and Chi-square test. Fisher’s exact test was used when there were fewer than five units in any of the classes. All of the continuous variables with normal distribution were presented as mean ± SD. Measurements of cffDNA were logarithmically transformed (to base 10) to remove skewness; all analyses presented used the logged values. Since the non-parametrically distributed, the log10 cffDNA, HCG and AFP concentrations were presented as median and interquartile range and the difference between two groups was analyzed by using the Mann–Whitney U test. Detection rate and false-positive rate were calculated for cffDNA and HCG together and cffDNA alone with a univariable receiver operating characteristic (ROC) curve. Finally, the correlation of the cffDNA levels with two prenatal screening markers: HCG and AFP, gestation weeks at delivery and babies’ birth weight were determined using the Spearman rank. A two-sided *p* value less than 0.05 was considered statistically significant.

## 4. Conclusions

In conclusion, our findings indicate that cffDNA in maternal serum is a potential noninvasive marker for EOPE with high sensitivity and specificity. It was strongly correlated with gestation weeks at delivery and babies’ birth weight. The increased level of cffDNA correlated significantly with the increased HCG level. Therefore, our study may help in providing indirect clues to the underlying hypoxic condition in the placenta in EOPE. However, our cffDNA is gender dependent, future research needs to identify markers that would allow monitoring of all pregnancies. Our study warrants further investigation with a large sample size, especially for EOPE.

## Figures and Tables

**Table 1:** Maternal epidemiological characteristics.

	Case (*n* = 30)	Control (*n* = 867)	*p*-values
Maternal age, y	30.47 ± 5.31	27.48 ± 4.70	0.001 *
Gestational weeks at enrollment, w	17.99 ± 1.17	17.87 ± 1.24	0.617
Pregestational body mass index, kg/m^2^	23.49 ± 3.57	21.30 ± 2.76	0.002 *
Nulliparous	80.0%	88.5%	0.158
Previous abortion	6.7%	0.9%	0.003 *
Family history of hypertension	63.3%	18.9%	<0.001 *
Medical history of hypertension, diabetes and nephritis	10.0%	0.3%	<0.001 *

* There is a significant difference between the case and control.

**Table 2:** Epidemiological characteristics in 40 cases of pregnant women carrying male fetus.

	Case (*n* = 20)	Control (*n* = 20)	*p*-values
Maternal age, y	29.54 ± 5.07	28.27 ± 4.78	0.421
Gestational weeks at enrollment, w	18.06 ± 1.37	17.80 ± 0.99	0.488
Getational weeks at delivery, w	35.66 ± 3.00	39.96 ± 0.48	<0.001 *
Birth weight, kg	2.79 ± 0.81	3.50 ± 0.22	0.001 *
Intrauterine growth restriction (IUGR)	10%	0%	0.487

* There is a significant difference between the case and control.
